# Knowledge, Attitudes and Perceptions Regarding Basic Life Support Among Teachers in Training

**DOI:** 10.7759/cureus.6302

**Published:** 2019-12-06

**Authors:** Kehinde Ojifinni, Feroza Motara, Abdullah E Laher

**Affiliations:** 1 Emergency Medicine, University of the Witwatersrand, Johannesburg, ZAF

**Keywords:** basic life support, bls, cardiopulmonary resuscitation, cpr, teachers, learners, school, knowledge, attitude, perception

## Abstract

Background

Cardiac arrests may occur anytime, anywhere and to anyone including learners at schools. Teachers have a moral obligation to care for learners while on the school premises. Outcomes after cardiac arrest are better when the first-responder possesses adequate knowledge and skill in basic life support (BLS) and cardiopulmonary resuscitation (CPR). The aim of this study was to assess the knowledge, attitudes and perceptions of student-teachers pertaining to BLS.

Methods

This was a self-administered, questionnaire based, prospective and cross-sectional study of senior undergraduate student-teachers enrolled at a South African university. The study was conducted between 04 November 2017 and 18 February 2018.

Results

A total of 316 student-teachers, with a mean age of 21.8 ± 2.6 years completed the survey. Trauma-related emergencies, allergic reactions and breathing difficulties were witnessed during practice teaching sessions at various schools by 52.5% (n = 166), 36.4% (n = 115) and 32.9% (n = 104) of participants, respectively. The mean knowledge score pertaining to BLS was 4.0 ± 1.7 out of 12 points. Previous CPR training was associated with a good knowledge score (p = 0.005) and confidence in responding to an emergency (p = 0.005). Most of the participants (N = 288, 91.1%) had no formal training in CPR with more than three-quarters (76.4%) of them not knowing where to acquire training. Barriers to initiating CPR included fear of litigation (n = 264, 83.5%), injury to the victim (n = 238, 75.3%), presence of blood, vomitus or secretions (n = 206, 65.2%) and fear of contracting a disease (n = 186, 58.8%). Most (n = 255, 80.7%) respondents reported that they would perform CPR on a learner at school.

Conclusion

Student-teachers surveyed in this study displayed poor knowledge and perceptions but positive attitudes with regards to the practice of CPR and BLS. Consideration should be given to including formal CPR training as part of the curriculum for teachers in training.

## Introduction

Children spend a considerable amount of time on the school premises away from their parents and guardians. For these learners, the school is a ‘home away from home’ and is supposed to be a ‘safe haven’ that offers them the needed healthy environment to learn, play and interact with fellow learners [1]. Despite precautionary measures, medical emergencies may occur unexpectedly. Various medical emergencies requiring immediate intervention, such as trauma-related conditions, airway and breathing problems, severe allergic reactions, near-drowning, and seizures have been reported among learners in schools [2].

Basic life support (BLS) skills include recognition of cardiac arrest, activation of local emergency medical services (EMS), initiation of cardiopulmonary resuscitation (CPR) and the use of an automated external defibrillator (AED). Initially aimed at healthcare workers, BLS including CPR has evolved as a skill taught to laymen. Since survival has been shown to decrease by 7-10% for every minute delay in the initiation of CPR, bystander-initiated CPR with the early use of AED may help save lives [3]. In fact, the early initiation of bystander CPR in cases of witnessed cardiac arrest has been shown to improve survival outcomes by approximately 50% [4].

Teachers are responsible for the well-being of learners during school hours. In some regions school teachers are increasingly being relied upon to administer medication, treat minor injuries and handle emergencies occurring during school hours [5]. With an average nurse to school ratio of between 1:20-1:30 in South Africa, there is a definite deficiency in the availability of healthcare staff at schools. Lack of collaboration between the Department of Basic Education (through the teachers), the Department of Health (through the health workers) and the Department of Social Development has been suggested as a possible reason for the failure of implementation of a successful school health policy in South Africa [6].

Several international studies have reported on the knowledge and attitudes of teachers with regards to the practice of BLS and bystander CPR in the school setting [7,8]. However, there is paucity of local data. Hence, this survey was conducted to determine the knowledge, attitude and perception relating to BLS and CPR among student-teachers at a South African university.

## Materials and methods

The cross-sectional, questionnaire-based study was conducted between 04 November 2017 and 18 February 2018. The study population comprised a convenience sample of 321 senior undergraduate students (3rd and 4th year of study) that were enrolled under the Faculty of Education at tertiary institute in South Africa. The final questionnaire (see appendix) was modified after being piloted on 17 students based at another institute in South Africa during September 2017. First- and second-year students were excluded from study participation since they were less likely to have sufficient experience in teaching practice.

The questionnaire comprised a total of 44 questions across four categories that included socio-demographic characteristics, medical emergencies encountered in the schools, knowledge of BLS as well as attitudes and perceptions relating to BLS and emergency care. The section relating to knowledge of BLS comprised 12 multiple choice questions, with a score of 6 and above being considered as a good knowledge score.

Ethics approval and permission to conduct the study was granted by the Human Research Ethics Committee (Medical) of the University (certificate clearance no. M170336) and the office of the Registrar respectively. Informed consent was obtained once participants had been given the opportunity to read through the study information sheet and agreed to participate in the study. Confidentiality was maintained at all times.

Collected data that was captured into an electronic data spread sheet (Microsoft® Excel®) and thereafter subjected to analysis. A descriptive analysis of the data is presented in the results section. Where appropriate, frequencies and proportions of various variables have been presented in tables and graphs. The Chi-squared test was used to test for significant differences between CPR trained and CPR untrained respondents. Significance was set at a p-value of <0.05.

## Results

**Figure 1:**
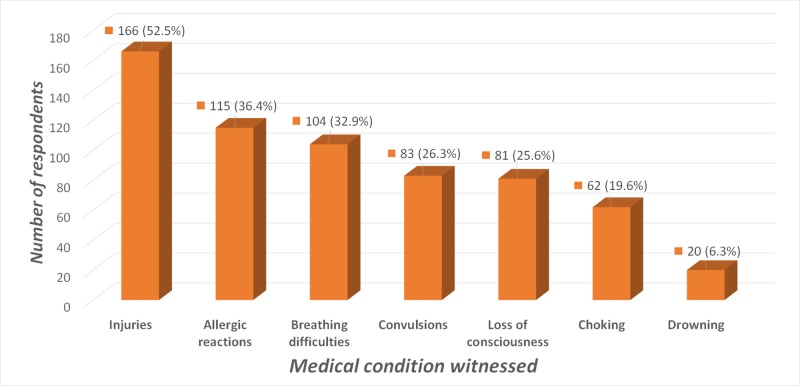
Medical conditions witnessed by respondents during practical teaching sessions at schools

Out of a total of 321 questionnaires administered, 316 (98.4%) were analyzed. Five (1.6%) questionnaires with incomplete data were excluded. The mean age of respondents was 21.8 ± 2.6 years. Most respondents were female (n = 194, 61.4%) and were aged between 20-29 years (n = 284, 89.9%). Figure 1 describes the number of respondents that had witnessed various medical emergencies during teaching practice sessions at schools. Injuries accounted for the majority of medical conditions witnessed (n = 166, 52.5%). Of note, 20 (6.3%) respondents indicated that they had witnessed an episode of drowning or near drowning.

With regards to the practice of BLS, the mean knowledge score was 4.0 ± 1.7 out of a maximum of 12 points. Only 56 (17.7%) respondents achieved a knowledge score of ≥6 points. Table 1 describes the proportion of correct answers for each of the BLS knowledge-based questions. Notably, only 35 (11.1%) respondents were aware of the first thing to look out for during a medical emergency, and just 110 (34.8%) were aware of the contact number of any EMS provider service in South Africa.

**Table 1:** Proportion of correct answers among respondents for each of the Basic Life Support knowledge-based questions

	n (%)
What is the first thing to look out for during a medical emergency?	35 (11.1)
What are the steps of Basic Life Support in an adult?	75 (23.7)
What is the most appropriate care for a child who is found unconscious in a swimming pool?	182 (57.6)
What is the chest compression to breath ratio for 1-rescuer child CPR?	69 (21.8)
What is the chest compression to breath ratio for 2-rescuer child CPR?	71 (22.5)
How often should roles be switched when performing 2-rescuer CPR?	61 (19.3)
What are the correct steps to operating an Automated External Defibrillator (AED) device?	52 (16.5)
Which is not a characteristic of high-quality CPR?	73 (23.1)
What is the current American Heart Association Basic Life Support sequence?	68 (21.5)
What are the signs of airway obstruction?	188 (59.5)
How would you check for responsiveness in a baby?	160 (50.6)
List the contact number of any Emergency Medical Services provider service in South Africa?	110 (34.8)

Table 2 describes the attitudes and perceptions of respondents pertaining to the practice of BLS. Most respondents (n = 286, 90.5%) indicated that CPR training should be mandatory for all teachers.

**Table 2:** Attitudes and perceptions of respondents pertaining to the practice of Basic Life Support

	n (%)
Have you ever provided emergency care to anyone?	76 (24.1)
Did you feel confident to handle an emergency during your teaching practice?	81 (25.6)
Have you ever received formal CPR training?	28 (8.9)
Should CPR training be mandatory for all teachers?	286 (90.5)
Would the inclusion of a CPR training course add extra work and demand to the current curriculum?	122 (38.6)

Alarmingly, only 28 (8.9%) participants had previously received formal training in CPR. Of the remainder, 253 (87.8%) indicated that they would like to undergo training in CPR. Compared to CPR untrained responders, a significantly greater proportion of respondents with CPR training had achieved a knowledge score of ≥6 (p = 0.005), had previously provided emergency care to anyone (p = 0.02) and were confident in dealing with medical emergencies during practice teaching sessions at schools (p = 0.005). These findings are described in Table 3.

**Table 3:** Comparison of knowledge scores and attitudes between CPR trained and CPR untrained respondents

	CPR trained (n = 28)	Not CPR trained (n = 288)	P-value
Knowledge score ≥ 6 out of 12	15 (53.6)	41 (14.2)	p = 0.005
Have you ever provided emergency care to anyone before?	12 (42.9)	64 (22.2)	p = 0.02
Are you confident in dealing with medical emergencies during practice teaching sessions at schools?	14 (50)	67 (23.3)	p = 0.005
Correct steps to operating an Automated External Defibrillator (AED) device?	3 (10.7)	18 (6.3)	p = 0.415

**Figure 2:**
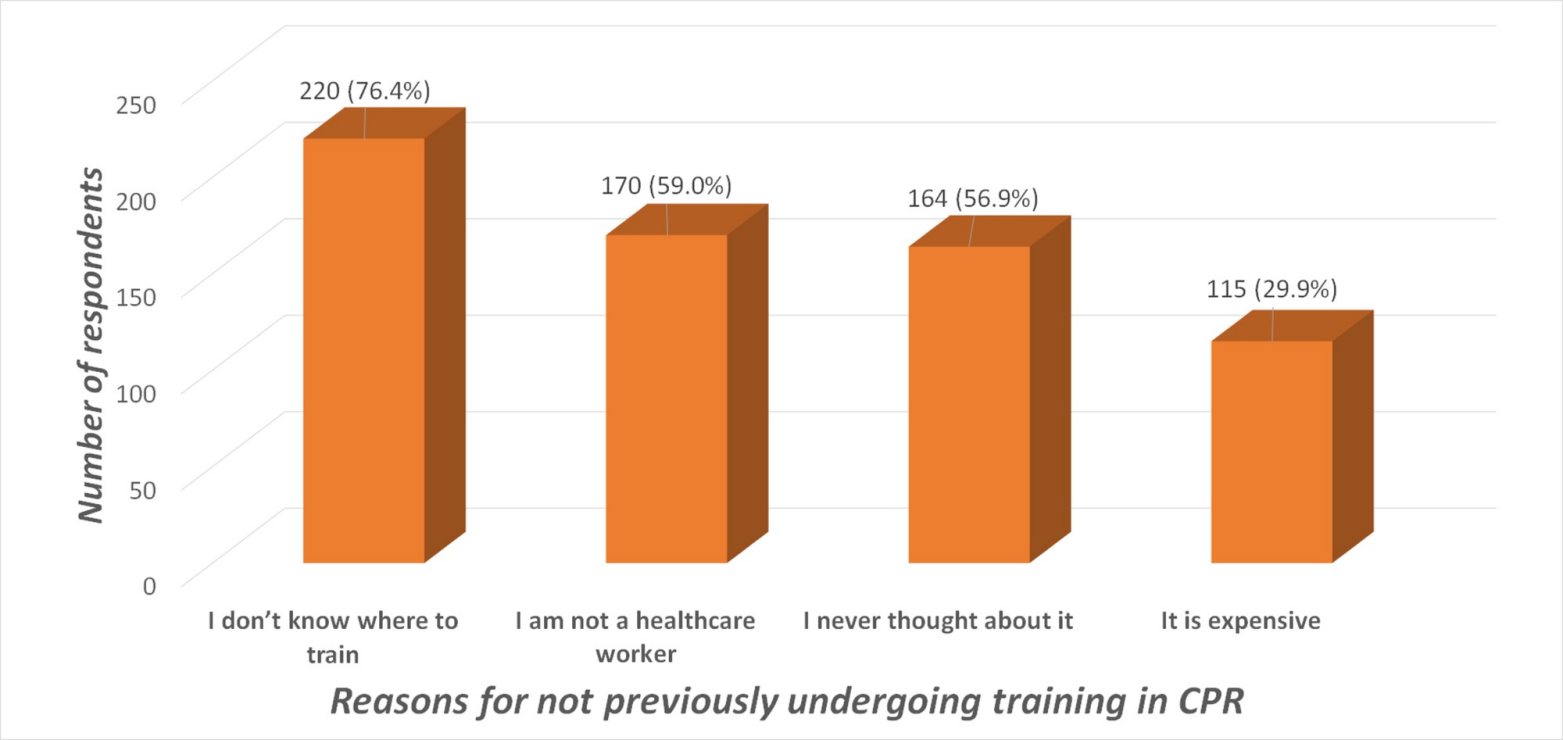
Reasons for not previously undergoing training in CPR among the two-hundred and eighty-eight CPR untrained respondents

Not knowing where to train was the commonest reason for not previously undergoing training in CPR. Other reasons are described in Figure 2.

Most (n = 255; 80.7%) participants reported that they would be willing to perform CPR on a learner at school requiring it. Table 4 describes the various barriers to providing CPR as well as the willingness of respondents to perform CPR in various categories of victims.

**Table 4:** Barriers to providing CPR and persons in whom respondents would be willing to provide CPR

	n (%)
BARRIERS TO PROVIDING CPR	
Fear of being sued if something goes wrong	264 (83.5)
Presence of blood, vomitus or secretions	206 (65.2)
Fear of injury to the victim	238 (75.3)
Fear of contracting a disease from the victim	186 (58.9)
Belief that someone else will do it	145 (45.9)
PERSONS IN WHOM RESPONDENTS WOULD BE WILLING TO PROVIDE CPR	
Immediate family member	285 (90.2)
An adult stranger	187 (59.2)
A child stranger	234 (74.1)
A learner in the school	255 (80.7)

## Discussion

Although an important component of healthy lifestyle, sports activities are a major cause of injury-related morbidity among school-age children [9]. In the current study, more than half the number of respondents (52.5%) reported that they had witnessed an injury to a learner at school. Statistics South Africa reported injury as the fourth leading cause of death in the 0-14 year age group, 10-25% of which occurred at schools [10]. A similar study in the USA identified injuries as the leading cause of death and disability among children, with 70% of injury-related deaths occurring in school children aged between 5-19 years [1].

Respiratory emergencies are also commonly seen among learners. Respiratory distress/failure has been reported as the leading cause of cardiac arrests in children [11]. About one-third (32.9%) of respondents in this study had seen a learner with a breathing difficulty (e.g., asthma). The Global Initiative for Asthma (GINA) reports that South Africa has the world’s fourth highest asthma-related mortality with more than 20% of school children across the region suffering from the condition [12]. Schools are also a high-risk setting for learners to manifest with potentially life threatening allergic reactions such as anaphylaxis [13]. In a study conducted among 132 children aged 3 to 19 years at the Johns Hopkins Hospital Pediatric Allergy Clinic, 18% of participants had experienced one or more allergic reaction whilst at school [14].

Various studies have shown that school teachers generally do not have adequate knowledge regarding BLS and CPR [7,15,16]. This is similar to findings in our study which showed a mean average BLS knowledge score of 4.0 ± 1.7 out of a maximum of 12 points. Although no previously published studies in South Africa have investigated the level of knowledge of BLS among teachers, a study conducted among preschool teachers in Turkey demonstrated poor knowledge of BLS with a mean knowledge score of 48.9% [16]. In the current study, a significant knowledge deficiency was noted in the correct steps to operating an AED. In the USA, use of an AED was associated with better survival after witnessed cardiac arrests at schools [17]. In fact, many schools in the USA have legislated the placement of AEDs at schools [18].

It is vital for every rescuer (layperson or professional) to look out for possible hazards before initiating CPR. In a study evaluating the need for BLS and first-aid training among early child development educators in Cape Town, South Africa, the lowest score was recorded for the identification of potential hazards [19]. Similarly, only 11.1% of respondents in this study correctly identified the first thing to look out for in an emergency.

The ability to easily remember and rapidly contact emergency services is crucial to the eventual outcome of a resuscitation effort. Only about one-third (34.8%) of the participants in this study were able to remember one or more contact numbers of local EMS provider services. This is in contrast to the study by Ghrayeb et al. in Palestine where only 18.1% of school teachers interviewed were unaware of the contact numbers for EMS provider services [8]. The lack of knowing the correct contact number in this study may be due to the fact that unlike other countries that only have a single universal contact number for medical emergencies, there are multiple universal emergency contact numbers in South Africa (e.g., 911, 112, 10177, 10111) that may have created confusion among responders. In addition, private EMS service providers in South Africa each have their own specific emergency contact numbers.

In the current study, the difference in BLS knowledge score between CPR-trained and CPR-untrained respondents was significant (p = 0.005). This is in contrast to the findings of a similar study on school teachers in Saudi Arabia that reported no significant difference between these two groups. A potential explanation for the low knowledge scores among the CPR-trained group in the Saudi Arabian study is that CPR training was completed more than two years prior to the study being conducted [20]. This highlights the ongoing need for CPR refresher training.

In this study, the willingness of respondents to initiate CPR was dependent on the relationship and age of the cardiac arrest victim (immediate family member > scholar > child stranger > adult stranger). This is in keeping with the findings of a study that was conducted in China where the majority (98.6%) of participants interviewed were willing to perform CPR on a family member compared to a stranger (76.3%) [21]. However, another Chinese study showed that CPR training was associated with a significant increase in willingness to perform CPR on a stranger [22].

Although bystander CPR has been shown to improve outcomes after cardiac arrest by two- to three-fold, very few victims actually receive bystander CPR. This has been attributed to various perceived barriers by laypersons to initiating CPR [23]. In this study, fear of litigation was reported as the most common reason for unwillingness to initiate CPR in a cardiac arrest victim. Internationally, there are Good Samaritan laws that protect lay persons who perform CPR on victims of cardiac arrest [24,25].

Fear of contracting a disease (58.9%) or contamination with blood, vomitus or other body fluids (65.2%) were other significant barriers to initiating CPR among study respondents. The high HIV prevalence in South Africa may be a likely explanation for this attitude [26]. In a study conducted in the United Kingdom, more than 80% of participants were willing to initiate full CPR on strangers in cardiac arrest. The proportion however fell to 40% in the presence of facial bleeding [27].

A limitation of this study is that participants were not qualified teachers but rather student-teachers whose experience may not be a true representation of the frequency of medical emergencies occurring in schools. Despite this, participants in this study nevertheless witnessed a substantial number of medical emergencies during practice teaching sessions in schools. Furthermore, since student-teachers are working under supervision, they may have also experienced some restrictions in their level of involvement in the overall care of the learners.

## Conclusions

Although attitudes towards BLS and CPR training were satisfactory in this study, participants displayed poor knowledge and perceptions. Given the willingness of participants to be trained, the South African Department of Health in conjunction with the Department of Education should consider implementing BLS and CPR training as part of the academic curriculum for student-teachers.

## Appendices

STUDY QUESTIONNAIRE

Age: ______________________________               Sex: Male / Female

Have you witnessed any of these medical conditions during your practice teaching sessions at any school?

Injury (e.g., cuts, broken limbs, bleeding, etc.)                        Yes / No

Loss of consciousness                                                             Yes / No

Choking                                                                                     Yes / No

Breathing difficulty (e.g., asthma attack)                                  Yes / No

Allergic reactions (to food, medication, insect bite, etc.)       Yes / No

Convulsions (seizures / fits)                                                     Yes / No

Drowning                                                                                  Yes / No

Knowledge about basic life support (Mark the most appropriate option)

1. What is the first thing to look out for during a medical emergency?

A. The victim’s level of consciousness

B. The victim’s breathing

C. The safety of the environment

D. The location of the Automated External Defibrillator (AED)

2. What are the steps of Basic Life Support in an adult?

A.    Assess the individual, give two rescue breaths, give a shock, and start Cardiopulmonary Resuscitation (CPR)

B.    Assess the individual, activate the Emergency Medical Services (EMS) and get an AED, check the pulse, and start CPR

C.    Check the pulse, give rescue breaths, assess the individual, and give a shock

D.    Assess the individual, start CPR, give two rescue breaths, and give a shock

3. What is the most appropriate care for a child who is found unconscious in a swimming pool?

A. Wrap the child in a warm blanket

B. Remove some aspirated water by chest thrusts

C. Shout at others for leaving the safety gate open

D. Start CPR and call an ambulance

4. What is the chest compression to breath ratio for 1-rescuer child CPR?

A.    30:1

B.    30:2

C.    15:1

D.    15:2

5. What is the chest compression to breath ratio for 2-rescuer child CPR?

A.    30:1

B.    30:2

C.    15:1

D.    15:2

6. How often should roles be switched when performing 2-rescuer CPR?

A.    After every cycle of CPR

B.    After every 2 cycles of CPR

C.    After every 5 cycles of CPR

D.    After every 10 cycles of CPR

7. What are the correct steps to operating an Automated External Defibrillator (AED) device?

A.    Check pulse, analyze rhythm and deliver a shock

B.    Attach electrodes pads, power on the AED, deliver a shock, and analyze the rhythm

C.    Power on the AED, attach electrode pads, deliver a shock, and analyze the rhythm

D.    Attach electrode pads, power on the AED, analyze the rhythm, clear the individual, and deliver a shock

8. Which is not a characteristic of high-quality CPR?

A.    Starting chest compressions within 10 minutes of recognition of cardiac arrest

B.    Push hard and fast

C.    Allow adequate chest recoil

D.    Minimizing interruptions

9. What is the current American Heart Association Basic Life Support sequence?

A.    Airway, Breathing, Compression

B.    Airway, Breathing, Check pulse

C.    Chest compression, Airway, Breathing

D.    None of the above

10. What are the signs of airway obstruction?

A.    Poor air exchange

B.    High-pitched noise while inhaling

C.    Inability to speak

D.    All of the above

11. How would you check for responsiveness in a baby?

A.    Shake the baby

B.    Shout at the baby

C.    Flick the bottom of the foot

D.    Tap the baby’s shoulders

12. List the contact number of any Emergency Medical Services provider service in South Africa?

_______________________________________________________________________

Attitude and perception about Basic Life Support

Have you ever received formal CPR training?                                                                                                 Yes / No

If no, what are the give reason:

a.    I am not a health care provider                                                                                                                   Yes / No

b.    I never thought about it                                                                                                                                Yes / No

c.    It costs money                                                                                                                                               Yes / No

d.    I do not know where to have the training                                                                                                   Yes / No

e.    Other reasons (specify): _________________________________________________________________

Would you like to have CPR training?                                                                                                                 Yes / No

Have you ever provided emergency care to anyone before?                                                                          Yes / No

Are you confident in dealing with medical emergencies during practice teaching sessions at schools?     Yes / No

Do you think CPR training should be mandatory for all teachers?                                                                    Yes / No

Do you consider CPR training an extra work and demand to the academic curriculum?                                Yes / No

Which of these will you consider a barrier to you providing CPR to an unconscious person?

a.   Fear of being sued if it goes wrong                                                                                                               Yes / No

b.   Presence of blood or vomit                                                                                                                            Yes / No

c.   Fear of injury to the victim                                                                                                                              Yes / No

d.   Fear of contracting disease from the victim                                                                                                  Yes / No

e.   Belief that someone else will do it                                                                                                                 Yes / No

Will you provide CPR to a victim if he or she is:

a.   A learner in your school                                                                                                                                  Yes / No

b.   An immediate family member                                                                                                                         Yes / No

c.  A stranger that is an adult                                                                                                                                Yes / No

d.  A stranger that is a child                                                                                                                                  Yes / No

Thank you for your participation
